# Supplementary material for: Acute kidney injury associated with COVID-19: A retrospective cohort study
Source: PLoS Med. 2020 Oct 30;17(10):e1003406. doi: 10.1371/journal.pmed.1003406 (PMC7598516; doi:10.1371/journal.pmed.1003406)
Supplement: S1 Text — (DOCX) [file pmed.1003406.s001.docx]

| **FULL/ LONG TITLE OF THE STUDY**  Acute Kidney Injury In corona virus infection disease (COVID-19) in Derby, United Kingdom | |
| --- | --- |
| **SHORT TITLE**  Acute Kidney Injury In COVID-19 | |
| **Version and Date of Protocol:** | v1.1 27/May/2020 |
| **Sponsor:** | University Hospitals of Derby & Burton NHS Foundation Trust |
| **Chief Investigator:** | Dr. Nitin V Kolhe |
| **Local Reference:** | UHDB/2020/050 |
| **IRAS Number:** | 284091 |
| **ISRCTN/ ClinicalTrials.gov number:** |  |
| **Funder(s):** | None |
| **This protocol has regard for the HRA guidance** | |

**Confidentiality Statement**

This document contains confidential information that must not be disclosed to anyone other than the Sponsor, the Investigator Team, host NHS Trust, regulatory authorities, and members of the Research Ethics Committee.

# SIGNATURE PAGE

The undersigned confirm that the following protocol has been agreed and accepted and that the Chief Investigator agrees to conduct the study in compliance with the approved protocol and will adhere to the principles outlined in the Declaration of Helsinki, the Sponsor’s SOPs, and other regulatory requirement.

I agree to ensure that the confidential information contained in this document will not be used for any other purpose other than the evaluation or conduct of the investigation without the prior written consent of the Sponsor.

I also confirm that I will make the findings of the study publically available through publication or other dissemination tools without any unnecessary delay and that an honest accurate and transparent account of the study will be given; and that any discrepancies from the study as planned in this protocol will be explained.

**Protocol v 1.1 27/May/2020 authorisation signatures:**

| 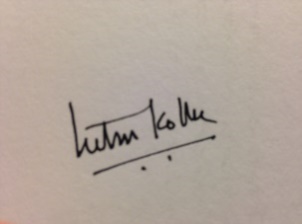**Chief Investigator:** | | | |
| --- | --- | --- | --- |
| Signature: | ………………………………… |  | Date:  27/MAY/2020 |
| Name (please print): | Dr. Nitin Kolhe |  |  |
|  | | | |

# KEY STUDY CONTACTS

| Chief Investigator: | Nitin V Kolhe; 01332788266; [nitin.kolhe@nhs.net](mailto:nitin.kolhe@nhs.net) |
| --- | --- |
| Co-Investigator(s): | Maarten W Taal; 01332789344; [maarten.taal1@nhs.net](mailto:maarten.taal1@nhs.net) |
| Sponsor: | University Hospitals of Derby & Burton NHS Foundation Trust  Royal Derby Hospital  Uttoxeter Road  Derby, DE22 3NE  01332 724639  [UHDB.sponsor@nhs.net](mailto:UHDB.sponsor@nhs.net) |
| Funder(s): | None |

# STUDY SUMMARY

| **Study Title:** | Acute Kidney Injury In COVID-19 in Derby, United Kingdom |
| --- | --- |
| **Local Study Reference:** | UHDB/2020/050 |
| **Study Design:** | Retrospective cohort study |
| **Study Participants:** | All patients suspected of COVID-19 admitted to UHDB NHS Trust between |
| **Planner Number of Sites:** | 1 |
| **Planned Sample Size:** | 330 AKI patients |
| **Planned Start Date:** | 13th May 2020 |
| **Planned Study End Date:** | 31^st^ May 2021 |
| **Research Question/ Aims:** | Epidemiology of COVID-19 patients who developed AKI in hospitalised patients in Derby, United Kingdom. |

# ROLES & RESPONSIBILITIES

**Sponsor**

The Sponsor, University Hospitals of Derby & Burton NHS Foundation Trust, take on overall responsibility for appropriate arrangements being in place to set up, run and report the research project. A Division of Responsibilities between the CI and Sponsor outlines the duties that have been delegated to the CI for this study.

**Study Management Committees**

No formal study management committees are required according to the Sponsor’s risk assessment for the study. The CI will provide status updates to the Sponsor according to their requirements.

Protocol Contributors

A number of protocol contributors have been involved in the development of this protocol, these include the Chief and the co-Investigator.

Protocol contributors are responsible for inputting into the design of the study, ensuring that it is designed transparently and efficiently.

Contents

[SIGNATURE PAGE 2](#_Toc39734959)

[KEY STUDY CONTACTS 3](#_Toc39734960)

[STUDY SUMMARY 4](#_Toc39734961)

[ROLES & RESPONSIBILITIES 5](#_Toc39734962)

[LIST OF ABBREVIATIONS 8](#_Toc39734963)

[STUDY FLOW CHART 9](#_Toc39734964)

[1. BACKGROUND 10](#_Toc39734965)

[2. RATIONALE 10](#_Toc39734966)

[3. OBJECTIVES AND OUTCOME MEASURES/ ENDPOINTS 10](#_Toc39734967)

[3.1. Objectives 10](#_Toc39734968)

[3.2. Outcome 11](#_Toc39734969)

[4. STUDY DESIGN 11](#_Toc39734970)

[5. STUDY SETTING 11](#_Toc39734971)

[6. ELIGIBILITY CRITERIA 11](#_Toc39734972)

[6.1. Inclusion Criteria 11](#_Toc39734973)

[6.2. Exclusion Criteria 11](#_Toc39734974)

[7. STUDY PROCEDURES 11](#_Toc39734975)

[7.1. Recruitment 11](#_Toc39734976)

[7.1.1. Patient Identification 11](#_Toc39734977)

[7.1.2. Screening 12](#_Toc39734978)

[7.2. Study Assessments 12](#_Toc39734979)

[7.3. End of Study 12](#_Toc39734980)

[8. DATA HANDLING 12](#_Toc39734981)

[8.1. System and Compliance 12](#_Toc39734982)

[8.2. Source Data 13](#_Toc39734983)

[8.3. Data Workflow 13](#_Toc39734984)

[8.4. Data Access and Security 13](#_Toc39734985)

[8.5. Archiving 13](#_Toc39734986)

[9. STATISTICS AND DATA ANALYSIS 13](#_Toc39734987)

[9.1. Sample Size Calculation 13](#_Toc39734988)

[9.2. Statistical Analysis 13](#_Toc39734992)

[9.2.1. Summary of Baseline Data and Flow of Patients 14](#_Toc39734993)

[9.2.2. Outcome Analysis 14](#_Toc39734994)

[9.2.3. Interim Analysis and Criteria for the Premature Termination of the Study 14](#_Toc39734995)

[9.2.4. Analysis Groups 14](#_Toc39734996)

[9.3. Procedure(s) to Account for Missing or Spurious Data 14](#_Toc39734997)

[10. MONITORING, AUDIT & INSPECTION 14](#_Toc39734998)

[11. ETHICAL AND REGULATORY CONSIDERATIONS 15](#_Toc39734999)

[11.1. Assessment and Management of Risk 15](#_Toc39735000)

[11.2. Peer review 15](#_Toc39735001)

[11.3. Public and Patient Involvement 15](#_Toc39735002)

[11.4. Research Ethics Committee (REC) & Regulatory Considerations 15](#_Toc39735003)

[11.5. Protocol Compliance / Non-compliance Reporting 15](#_Toc39735004)

[11.6. Notification of Serious Breaches to GCP and/or the Protocol 16](#_Toc39735005)

[11.7. Data Protection and Patient Confidentiality 16](#_Toc39735006)

[11.8. Financial and Other Competing Interests for the Chief Investigator, Principal Investigators at Each Site and Committee Members for the Overall Study Management 16](#_Toc39735007)

[11.9. Indemnity 16](#_Toc39735008)

[11.10. Amendments 16](#_Toc39735009)

[11.11. Access to Final Study Dataset 17](#_Toc39735010)

[12. DISSEMINATION POLICY 17](#_Toc39735011)

[12.1. Dissemination Policy 17](#_Toc39735012)

[12.2. Authorship Eligibility Guidelines and any Intended Use of Professional Writers 17](#_Toc39735013)

[13. APPENDICES 19](#_Toc39735014)

[13.1. Appendix 2 – Amendment History 19](#_Toc39735015)

# LIST OF ABBREVIATIONS

| AE | Adverse Event |
| --- | --- |
| CI | Chief Investigator |
| CRF | Case Report Form |
| DMEC | Data Monitoring and Ethics Committee |
| GCP | Good Clinical Practice |
| GMP | Good Manufacturing Practice |
| ICF | Informed Consent Form |
| ICH | International Conference on Harmonisation of technical requirements for registration of pharmaceuticals for human use. |
| ISF | Investigator Site File |
| ISRCTN | International Standard Randomised Controlled Trials |
| NHS R&D | National Health Service Research & Development |
| PI | Principal Investigator |
| PIC | Participant Identification Centre |
| PIS | Participant Information Sheet |
| QA | Quality Assurance |
| QC | Quality Control |
| RCT | Randomised Control Trial |
| REC | Research Ethics Committee |
| SAE | Serious Adverse Event |
| SDV | Source Data Verification |
| SOP | Standard Operating Procedure |
| TMG | Trial Management Group |
| TSC | Trial Steering Committee |
| TMF | Trial Master File |

# STUDY FLOW CHART


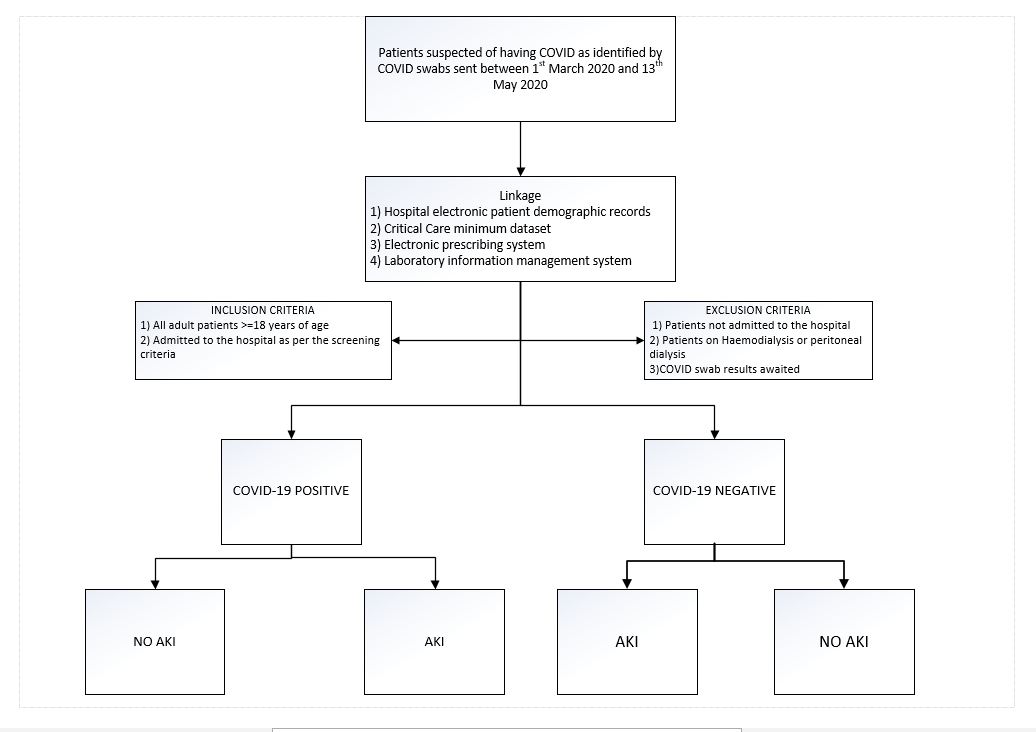


**STUDY PROTOCOL**

# BACKGROUND

On 31^st^ December 2019, a cluster of pneumonia cases were detected in Hubei province of China where the cause was unclear. The cause for these cases was subsequently identified as a virus which was referred to as SARS-CoV-2, and the associated disease as COVID-19.^1^ This evolved rapidly into a global pandemic with first case reported in UK as early as on 30^th^ January 2020.^2^ As of 30^th^ April 2020, there have been 3.05 million laboratory confirmed cases world-wide with 161,145 cases in United Kingdom in 539,768 cases tested for COVID-19.^3^ The epidemiology of COVID-19 has differed in China, US and Italy which have different demographic characteristics, testing methods and different ways to curb the spread of infection which can be altered by many things, including behaviour, and the stringent social distancing measure.^4-7^ Though the pandemic started in China, as of 30^th^ April 2020, it has reported only 58.32 cases per million people (pmp) as compared to United kingdom which has reported 2433.8 cases pmp.^3^ This has resulted in different epidemiology of COVID-19 and organ system involvement. Lung involvement in the form of alveolar haemorrhages and respiratory failure has been well document in patients with COVID-19 and has been focus of attention, however, other organs are also affected in COVID-19, but there has been limited information kidney involvement in COVID patients.^5,8^ In initial reports, the acute kidney injury (AKI) incidence in COVID-19 has ranged from 5% to 29% with substantial variation between centres,

possibly due to differences in population demographics and risk factors for AKI.^1,2,5-7^ Initial reports on a small number of patients suggest that SARS-CoV-2 may have a specific effect on the kidneys but it is not yet clear to what extent COVID-19 increases the risk of AKI or how COVID-19 impacts outcomes after AKI.^8,9^

# RATIONALE

The aim of this study is to describe epidemiology of acute kidney injury developing in COVID-19 disease.

Urinary sediment abnormality has been described in COVID-19 patients, but this has also been reported in many critically unwell patients.^9^ In the previous SARS pandemic the reported incidence of AKI was 6.7% with a high mortality of over 90%.^10^ However, SARS-CoV-2 is a is a novel betacoronavirus belonging to the sarbecovirus subgenus of Coronaviridae family and its effects on kidneys are not well known. Careful monitoring of kidney disease and renal replacement therapy (RRT) is extremely important to manage resources which may become scarce during this pandemic.

# OBJECTIVES AND OUTCOME MEASURES/ ENDPOINTS

## Objectives

Primary objective

The primary objective is to describe the epidemiology of acute kidney injury associated with COVID-19 in patients admitted to hospital.

Secondary objectives:

To investigate the association between patient characteristics and patient outcomes in patients admitted with COVID-19.

## Outcome

The primary outcome is acute kidney injury in patients with COVID-19 disease.

Secondary outcomes will include all-cause mortality, need for ventilatory support, admission to ICU, length of stay.

# STUDY DESIGN

This is a retrospective, cohort study of patient data collected during routine care in the hospital settings.

# STUDY SETTING

This is a retrospective, single centre, cohort study of patients with suspected COVID-19 admitted in UHDB Foundation Trust and who had COVID-19 swab sent between 5^th^ March 2020 and 13^th^ May 2020.

# ELIGIBILITY CRITERIA

## Inclusion Criteria

All patients who are admitted to the hospital with suspected COVID-19 infection between 1^st^ March 2020 and 13^th^ May 2020 and who meet following criteria will be included

1. Adult patients greater or equal to 18 years of age.
2. Swab results available for SARS CoV-19
3. Patients admitted to the hospital as per the national screening criteria

## Exclusion Criteria

The following patients will be excluded from the study

1. Paediatric patients as defined by age of <18 years of age
2. Swab results which are awaited
3. Patients on haemodialysis or peritoneal dialysis

# STUDY PROCEDURES

## Recruitment

### Patient Identification

As this is retrospective cohort study, patients will be identified from microbiology dataset which contains details of nasal and pharyngeal swabs sent for real time reverse transcriptase polymerase chain reaction (RT-PCR) for COVID-19.

Eligible patients will be identified from the hospital dataset, if they had COVID swabs sent for RT-PCR. The data in the microbiology dataset will be linked to hospital electronic patient records containing demographic details to obtain identifiable personal information. The dataset will be linked to other data sources like laboratory information management system, electronic patient prescribing and critical care minimum dataset.

### Screening

As this is a retrospective, cohort study, patients will not be screened by the investigators.

In UHDB, patients are suspected of having COVID-19 if the symptoms include fever greater than or equal to 37.8°C and at least one of the following which must be of acute onset: persistent cough (with or without sputum, hoarseness, nasal discharge or congestion, shortness of breath, sore throat, wheezing, sneezing). Patients are admitted to the hospital as per their clinical needs.

AKI will be identified using modified Kidney Disease Improving Global Outcomes (KDIGO) definition of AKI as identified by the NHS England’s algorithm.^11^ The algorithm compares the current measured serum creatinine from an individual patient against the baseline creatinine value defined as either the lowest in the last 7 days or a median of values from the preceding 8 to 365 days depending on availability of previous results stored in laboratory information management system in real time. Urine output will not be used in generating the AKI alerts using NHS England’s AKI algorithm.

## Study Assessments

The data used to perform this study is from usual clinical practice in UHDB NHS Trust and as such all assessments and follow up are as per clinical needs.

## End of Study

The end date for the study will be 31^st^ May 2021

# DATA HANDLING

## System and Compliance

The data will be extracted in excel format and will be stored on encrypted and password protected computer. The data will be checked for errors and duplicates and once accuracy is confirmed, a final version without patient identifiable will be used to analysis on a statistical software.

## Source Data

The data source will be the UHDB NHS Trust’s data warehouse. Data quality reports and checks are completed at various stages in the cleaning and processing cycle, and any duplication is removed.

## Data Workflow

The data will be extracted from data warehouse and will be linked to laboratory information management system, electronic patient records, electronic prescribing cohort and critical care minimum dataset. The data will be hosted on a Trust computer with password protected encryption. The data quality will be checked to ensure validity and the chief investigator will be responsible for the data security.

## Data Access and Security

Data will be stored on an encrypted computer of the chief investigator which is password protected. Direct access will be granted to authorised representatives from the Sponsor, host institution and the regulatory authorities to permit study-related monitoring, audits and inspections. Data will not be transferred outside of the UK.

## Archiving

At the end of the study, following completion of the end of study report, UHDB will securely archive all centrally held study related documentation for a minimum of 5 years. At the end of the defined archive period arrangements for confidential destruction will be made. The data and all essential documents relating to the study will be retained securely for a minimum of 5 years after the end of study, and in accordance with national legislation. All archived documents will continue to be available for inspection by appropriate authorities upon request.

# STATISTICS AND DATA ANALYSIS

Descriptive analysis, percentages and 95% Confidence Intervals will be used initially for estimating the incidence of acute kidney injury in COVID-19 positive patients. Chi-squared or Fisher’s Exact tests will also be performed for categorical variables. Multivariable logistic regression analysis will be used to assess the impact of various baseline variables on acute kidney injury and secondary outcomes including mortality.

## Sample Size Calculation

This is a retrospective, non-CTIMP cohort study and randomization or related processes are not applicable. As of 4^th^ May 2020, there have been 311 cases of acute kidney injury in UHDB NHS Trust

## Statistical Analysis

### Summary of Baseline Data and Flow of Patients

Descriptive statistics will be presented to summarize the distribution of baseline variables across each of the groups. The continuous baseline variables will be reported with means & 95% confidence intervals (95% CI), if shown to be normally distributed will be reported with medians & Interquartile Ranges (IQR). The categorical variables will be reported with frequencies & percentages.

### Outcome Analysis

Univariate analysis will be performed to ascertain any significant association of baseline variables with mortality in patients with COVID-19 disease. Any variables with significant association in univariate analysis will be entered in multivariable logistic regression analysis to ascertain the effects of age, gender, ethnicity, comorbidities, medications, use of critical care and acute kidney injury on the likelihood of in-hospital death. A multivariable logistic regression will be performed to ascertain the effects of variable found to be significant in univariate analysis on AKI as an outcome in COVID-19 disease. All tests will be two-tailed, and p<0.05 will be considered significant. Analysis will be performed on IBM SPSS Statistics for Mac, Version 24.0

### Interim Analysis and Criteria for the Premature Termination of the Study

This is not applicable to a retrospective cohort study

### Analysis Groups

Descriptive analysis will be performed in patients with COVID-19 disease between patients who didn’t have AKI with those who had AKI in COVID-19 disease, between AKI in COVID-19 disease and a control group of patients with AKI with no COVID-19 disease and between survivors and non-survivors in COVID-19 disease. For this, categorical variables will be described as frequency and percentages. Continuous variables will be described as mean with standard deviation or median with inter-quartile range. Means for continuous variable will be compared using t-test if the data is normally distributed otherwise Mann Whitney test will be used. Proportion from categorical variables will be compared with chi square test.

## Procedure(s) to Account for Missing or Spurious Data

No imputation was made for missing data.

# MONITORING, AUDIT & INSPECTION

The investigator will permit study-related monitoring, audits and inspections by the Sponsor as required. This study will not be routinely monitored by the Sponsor, in accordance to the low risk nature of the study, but may be subject to audit for the QA team within the R&D department.

.

# ETHICAL AND REGULATORY CONSIDERATIONS

## Assessment and Management of Risk

As part of the Sponsor’s review process for all research projects, the study has been risk assessed according to their SOP. This study has been assessed as low risk.

## Peer review

The study protocol was peer reviewed by Prof. Maarten Taal, co-investigator of the study.

## Public and Patient Involvement

Patient has been involved in the design of protocol and will be involved in result and dissemination of the findings

## Research Ethics Committee (REC) & Regulatory Considerations

The study will be conducted in compliance with the approved protocol and the Declaration of Helsinki. The protocol and all related documentation (e.g. informed consent form, participant information sheet, questionnaires) have been reviewed and received approval by a Research Ethics Committee (REC). The investigator will not begin any participant activities until approval from the HRA and REC has been obtained and documented. All documentation and correspondence must be retained in the trial master file/investigator site file. Substantial amendments that require HRA and REC (where applicable) review will not be implemented until the HRA and REC grants a favourable opinion (with the exception of those necessary to reduce immediate risk to participants).

It is the responsibility of the CI to ensure that an annual progress report (APR) is submitted to the REC within 30 days of the anniversary date on which the favourable opinion was given, annually until the study is declared ended. The CI is also responsible for notifying the REC of the end of study (see Section 6.9) within 90 days. Within one year of the end of study, the CI will submit a final report with the results, including any publications/abstracts to the REC.

Before any site can enroll a patient into the study confirmation of capacity must be sought from the site’s research and development (R&D) department. In addition for any amendment that will potentially affect the site’s permission, the research team must confirm with the site’s R&D department that permission is ongoing (Section 11.10).

## Protocol Compliance / Non-compliance Reporting

The investigator is responsible for ensuring that the study is conducted in accordance with the procedures described in this protocol. Prospective, planned deviations and/or waivers to the protocol are not acceptable, however accidental protocol deviations (non-compliances) may happen and as such these must be recorded. Non-compliances should be recorded in the CRF and/or a non-compliance log kept in the ISF. All non-compliances should be reviewed and assessed by the PI (or appropriately delegated individual) to determine if they meet the criteria of a “serious breach” (Section 12.6). Non-compliances which are found to frequently recur are not acceptable, will require immediate action, and could potentially be classified as a serious breach.

## Notification of Serious Breaches to GCP and/or the Protocol

A “serious breach” is a departure from the protocol, Sponsor procedures (i.e. SOPs), or regulatory requirements which is likely to effect to a significant degree –

- 1. The safety or physical or mental integrity of the subjects of the study; or
  2. The scientific value of the study.

If the PI (or delegate) is unsure if a non-compliance meets these criteria, they should consult the Sponsor for further guidance.

If a serious breach is identified the investigator should notify the Sponsor immediately (i.e. within 1 working day) using the ‘Non-CTIMP Notification of a Serious Breach’ form. The report will be reviewed by the Sponsor and CI, and where appropriate, the Sponsor will notify the REC within 7 calendar days of being made aware of the breach.

## Data Protection and Patient Confidentiality

The study will be conducted in accordance with the Data Protection Act 2018. The investigator will ensure that participant’s anonymity is maintained throughout the study and following completion of the study. Once the final dataset has been created the data will be pseudo-anonymised, with each patient given a study ID with no patient identifiable data

All documents will be stored securely with access restricted to study staff and authorised personnel.

Nitin V Kolhe will act as the custodian of the data generated in the study.

## Financial and Other Competing Interests for the Chief Investigator, Principal Investigators at Each Site and Committee Members for the Overall Study Management

The researchers declare no conflict of financial or other competing interest

## Indemnity

As UHDB is acting as the research Sponsor for this study, NHS indemnity applies. NHS indemnity provides cover for legal liabilities where the NHS has a duty of care. Non-negligent harm is not covered by the NHS indemnity scheme. UHDB, therefore, cannot agree in advance to pay compensation in these circumstances. In exceptional circumstances an ex-gratia payment may be offered.

## Amendments

If changes to the study are required these must be discussed with the Sponsor, who is responsible for deciding if an amendment is required and if it should be deemed substantial or non-substantial. Substantial amendments will be submitted to the relevant regulatory bodies (REC, HRA) for review and approval. The amendments will only be implemented after approval and a favourable opinion has been obtained. Non-substantial amendments will be submitted to the HRA for their approval/ acknowledgment. Amendments will not be implemented until all relevant approvals are in place.

## Access to Final Study Dataset

Dr. Nitin Kolhe will have the access to final dataset which will be pseudo-anonymised.

# DISSEMINATION POLICY

## Dissemination Policy

The data is owned by UHDB NHS Trust and on completion of the study, the data will be analysed and tabulated and a Final Study Report prepared.

- The full study report can be accessed from the chief investigator.
- Participating investigators will have rights to publish any of the study data after discussion with the chief investigator.
- UHDB NHS Trust who sponsored the study will be acknowledged within the publications.
- The study protocol, full study report, pseudo-anonymised participant level dataset, and statistical code for generating the results will be made publicly available on clinicaltrials.gov after ethics approval.
- The study will be reported using the STROBE checklist for observational studies
- Limitation of the study includes –
  - Retrospective nature of the study
  - No urinary chemistry will be available
  - Sampling bias as ethnicity in Derbyshire & Staffordshire may have lower proportion of black population and the results may not be generalizable to other areas
  - Long-term effect of COVID-19 on AKI wont be available
  - Unmeasured confounders was dealt with COVID negative AKI control group where exposure to COVID-19 is not expected to be related to the outcome. This would address the potential magnitude of uncontrolled confounding

## Authorship Eligibility Guidelines and any Intended Use of Professional Writers

Authorship will be provided as per the International Committee of Medical Journal Editors definition of authorship criteria for manuscripts submitted for publication.

REFERENCES

1. Huang C, Wang Y, Li X, et al. Clinical features of patients infected with 2019 novel coronavirus in Wuhan, China. Lancet 2020;395:497-506.

2. <https://coronavirus.data.gov.uk/>. 2020. (Accessed 2nd May 2020, 2020,

3. <https://www.ecdc.europa.eu/en/geographical-distribution-2019-ncov-cases>. 2020. (Accessed 2nd May 2020, 2020,

4. Bi Q, Wu Y, Mei S, et al. Epidemiology and transmission of COVID-19 in 391 cases and 1286 of their close contacts in Shenzhen, China: a retrospective cohort study. The Lancet Infectious Diseases 2020.

5. Chen N, Zhou M, Dong X, et al. Epidemiological and clinical characteristics of 99 cases of 2019 novel coronavirus pneumonia in Wuhan, China: a descriptive study. Lancet 2020;395:507-13.

6. Grasselli G, Zangrillo A, Zanella A, et al. Baseline Characteristics and Outcomes of 1591 Patients Infected With SARS-CoV-2 Admitted to ICUs of the Lombardy Region, Italy. JAMA 2020.

7. Richardson S, Hirsch JS, Narasimhan M, et al. Presenting Characteristics, Comorbidities, and Outcomes Among 5700 Patients Hospitalized With COVID-19 in the New York City Area. JAMA 2020.

8. Guan WJ, Ni ZY, Hu Y, et al. Clinical Characteristics of Coronavirus Disease 2019 in China. N Engl J Med 2020;382:1708-20.

9. Han SS, Ahn SY, Ryu J, et al. Proteinuria and hematuria are associated with acute kidney injury and mortality in critically ill patients: a retrospective observational study. BMC Nephrol 2014;15:93.

10. Chu KH, Tsang WK, Tang CS, et al. Acute renal impairment in coronavirus-associated severe acute respiratory syndrome. Kidney Int 2005;67:698-705.

11. <https://www.england.nhs.uk/wp-content/uploads/2014/06/psa-aki-alg.pdf>. 2015. (Accessed 3rd May 2020, 2020,

# APPENDICES

## Appendix 2 – Amendment History

| **Amendment No.** | **Protocol version no.** | **Date issued** | **Author(s) of changes** | **Details of changes made** |
| --- | --- | --- | --- | --- |
| 1 | 1.1 | 27/May/2020 | NK | Updates to study design and statistical analysis plan following peer reviewer comments.  Updates to the study dates to reflect actual dates. |

Detail all protocol amendments. Protocol amendments must be submitted to the Sponsor for approval prior to submission to the REC.
