# Supplementary material for: Acute kidney injury associated with COVID-19: A retrospective cohort study
Source: PLoS Med. 2020 Oct 30;17(10):e1003406. doi: 10.1371/journal.pmed.1003406 (PMC7598516; doi:10.1371/journal.pmed.1003406)
Supplement: S1 Table — (DOCX) [file pmed.1003406.s003.docx]

**S1 Table: Baseline characteristics between survivors and non-survivors with COVID-19**

|  |  |  |  |  |
| --- | --- | --- | --- | --- |
|  |  | Survivors  Number (%) | Non-survivors  Number (%) | p-value |
|  |  | 742 (63·9) | 419 (36·1) |  |
| **Demographics** |  |  |  |  |
| Age in years^Ϯ^ |  | 68·9 ± 17·2 | 77·7 ± 12·0 | <0·001 |
| Age group COVID | 18-64 | 266 (35·8 | 55 (13·1) | <0·001 |
|  | 65-84 | 331 (44·6) | 232 (55·4) |  |
|  | 85+ | 145 (19·5) | 132 (31·5) |  |
| Gender | Male | 394 (53·1) | 263 (62·8) | 0·002 |
| Ethnicity | White | 546 (73·6) | 330 (78·8) | 0·279 |
|  | Asian | 71 (9·6) | 25 (6·0) |  |
|  | Black | 14 (1·9) | 7 (1·7) |  |
|  | Not stated | 97 (13·1) | 49 (11·7) |  |
| Care home residence |  | 103 (13·9) | 80 (19·1) | 0·023 |
| **Comorbidities** |  |  |  |  |
| Myocardial infarction |  | 53 (7·1) | 66 (15·8) | 0·006 |
| Congestive cardiac failure |  | 94 (12·7) | 113 (27·0) | <0·001 |
| Peripheral vascular disease |  | 23 (3·1) | 43 (10·3) | <0·001 |
| Cerebrovascular disease |  | 66 (8·9) | 51 (12·2 | 0·084 |
| Dementia |  | 69 (9·3) | 95 (22·7) | <0·001 |
| Chronic lung disease |  | 172 (23·2) | 139 (33·2) | <0·001 |
| Connective tissue disorder |  | 42 (5·7) | 32 (7·6) | 0·211 |
| Diabetes with complications |  | 142 (19·1) | 113 (27·0) | 0·002 |
| Paraplegia |  | 16 (2·2) | 13 (3·1) | 0·332 |
| Chronic kidney disease |  | 89 (12·0) | 135 (32·2) | <0·001 |
| Chronic liver disease |  | 5 (0·7) | 12 (2·9) | <0·001 |
| Cancer |  | 42 (5·7) | 60 (14·3) | <0·001 |
| **Treatment** |  |  |  |  |
| ACEI or ARB use^ꙶ¥^ |  | 108 (14·6) | 50 (11·9) | 0·250 |
| Mechanical ventilation |  | 40 (5·4) | 41 (9·8) | 0·006 |
|  |  |  |  |  |
| **Outcome** |  |  |  |  |
| AKI |  | 120 (16·2) | 184 (43·9) | <0·001 |
| Length of stay (days)^Ϯ^ |  | 7·6 ± 10·9 | 7·8 ± 9·8 | 0·765 |

^¥^Angiotensin converting enzyme or angiotensin receptor blocker

^Ϯ^ Mean ± standard deviation
