# Supplementary material for: Acute kidney injury associated with COVID-19: A retrospective cohort study
Source: PLoS Med. 2020 Oct 30;17(10):e1003406. doi: 10.1371/journal.pmed.1003406 (PMC7598516; doi:10.1371/journal.pmed.1003406)
Supplement: S2 Table — (DOCX) [file pmed.1003406.s004.docx]

**S2 Table: Univariate analysis of risk factors in survivors versus non-survivors in patients who developed AKI**

|  |  | Survivors  Number (%) | Non- survivors  Number (%) | p-value |
| --- | --- | --- | --- | --- |
| **Demographics** |  |  |  |  |
| Number of patients |  | 424 | 300 |  |
| Age in years^Ϯ^ |  | 71·8 ± 16·5 | 76·7 ± 12·6 | <0·001 |
| Age group (years) | 18-64 | 122 (28·8) | 48 (16·0) | <0·001 |
|  | 65-84 | 196 (46·2) | 164 (34·7) |  |
|  | 85+ | 106 (25·0) | 88 (29·5) |  |
| Gender | Male | 214 (50·5) | 179 (59·7) | 0·015 |
| Ethnicity | White | 360 (84·9) | 232 (77·3) | 0·153 |
|  | Asian | 17 (4·0) | 14 (14·7) |  |
|  | Black | 7 (4·0) | 7 (2·3) |  |
|  | Not stated | 34 (8·0) | 41 (13·7) |  |
| Care home residence |  | 65 (15·3) | 49 (16·3) | 0·756 |
| **Comorbidities** |  |  |  |  |
| Myocardial infarction |  | 32 (7·5) | 50 (16·7) | <0·001 |
| Congestive cardiac failure |  | 63 (14·9) | 107 (35·7) | <0·001 |
| Peripheral vascular disease |  | 17 (4·0) | 26 (8·7) | 0·011 |
| Cerebrovascular disease |  | 29 (6·8) | 32 (10·7) | 0·079 |
| Dementia |  | 36 (8·5) | 58 (19·3) | <0·001 |
| Chronic lung disease |  | 77 (18·2) | 89 (29·7) | <0·001 |
| Connective tissue disorder |  | 21 (5·0) | 25 (8·3) | 0·088 |
| Diabetes with complications |  | 72 (17·0) | 78 (26·0) | <0·001 |
| Paraplegia |  | 4 (0·9) | 9 (3·0) | 0·049 |
| Chronic kidney disease |  | 79 (18·6) | 109 (36·3) | <0·001 |
| Chronic liver disease |  | 9 (2·1) | 18 (6·0) | 0·009 |
| Cancer |  | 33 (7·8) | 47 (15·7) | <0·001 |
| **AKI characteristics** |  |  |  |  |
| Peak AKI | Stage 1 | 279 (65·8) | 147 (49·0) | <0·001 |
|  | Stage 2 | 78 (18·4) | 71 (23·7) |  |
|  | Stage 3 | 67 (15·8) | 82 (27·3) |  |
| Hospital AKI |  | 210 (49·5) | 171 (57·0) | 0·050 |
| AKI stage progression |  | 49 (11·6) | 78 (26·0) | <0·001 |
| **Treatment** |  |  |  |  |
| ACEI or ARB use^ꙶ¥^ |  | 71 (16·7) | 29 (9·7) | 0·006 |
| Need for intensive care |  | 50 (11·8) | 57 (19·0) | 0·008 |
| Mechanical ventilation |  | 41 (9·7) | 41 (13·7) | 0·097 |
| Renal replacement therapy |  | 12 (2·8) | 20 (6·7) | 0·017 |
| Renal support (days) ^Ϯ^ |  | 3·8 ± 8·6 | 2·7 ± 5·1 | 0·449 |
| **Outcome** |  |  |  |  |
| Length of stay (days)^Ϯ^ |  | 8·8 ± 13·8 | 9·4 ± 12·7 | 0·537 |

^¥^Angiotensin converting enzyme or angiotensin receptor blocker

^Ϯ^ Mean ± standard deviation
