# Supplementary material for: Acute kidney injury associated with COVID-19: A retrospective cohort study
Source: PLoS Med. 2020 Oct 30;17(10):e1003406. doi: 10.1371/journal.pmed.1003406 (PMC7598516; doi:10.1371/journal.pmed.1003406)
Supplement: S3 Table — (DOCX) [file pmed.1003406.s005.docx]

**S3 Table: Predictors of mortality in COVID-19 disease including Charlson's comorbidity score**

|  |  | Odds ratio (95% CI) | p-value |
| --- | --- | --- | --- |
| Age group | 18-64 | 1 (Ref) |  |
|  | 65-84 | 3·31 (2·18 , 5·03) | <0·001 |
|  | 85+ | 4·35 (2·69 , 7·06) | <0·001 |
| Gender | Male | 1·47 (1·11 , 1·95) | 0·007 |
| Ethnicity | White | 1 (Ref) |  |
|  | Asian | 1·16 (0·66 , 2·03) | 0·607 |
|  | Black | 1·02 (0·33 , 3·13) | 0·976 |
|  | Mixed | 1·92 (0·29 , 12·8) | 0·500 |
|  | Others | 1·95 (0·57 , 6·69) | 0·291 |
|  | Not stated | 0·97 (0·63 , 1·5) | 0·897 |
| Care home residence |  | 0·99 (0·68 , 1·43) | 0·946 |
| Mechanical ventilation |  | 3·60 (2·03 , 6·39) | <0·001 |
| ACEI or ARB use^ꙶ¥^ |  | 0·74 (0·5 , 1·11) | 0·142 |
| AKI |  | 3·25 (2·39 , 4·43) | <0·001 |
| Charlson's comorbidity score | 0 | 1 (Ref) |  |
|  | 1 | 1·99 (1·31 , 3·05) | <0·001 |
|  | 2 | 3·29 (2·12 , 5·1) | <0·001 |
|  | 3 | 3·93 (2·4 , 6·43) | <0·001 |
|  | 4 | 4·34 (2·55 , 7·41) | <0·001 |
|  | 5+ | 5·49 (3·51 , 8·61) | <0·001 |

^¥^ Angiotensin converting enzyme or angiotensin receptor blocker
