# Supplementary material for: Acute kidney injury associated with COVID-19: A retrospective cohort study
Source: PLoS Med. 2020 Oct 30;17(10):e1003406. doi: 10.1371/journal.pmed.1003406 (PMC7598516; doi:10.1371/journal.pmed.1003406)
Supplement: S4 Table — (DOCX) [file pmed.1003406.s006.docx]

**S4 Table: Predictors of mortality in AKI including Charlson's comorbidity score**

|  |  | Odds ratio (95% CI) | P-value |
| --- | --- | --- | --- |
| Age group | 18-64 | 1 (Ref) |  |
|  | 65-84 | 2·86 (1·69 , 4·83) | <0·001 |
|  | 85+ | 3·26 (1·79 , 5·96) | <0·001 |
| Gender | Male | 1·22 (0·86 , 1·74) | 0·270 |
| Ethnicity | White | 1 (Ref) |  |
|  | Asian | 1·30 (0·54 , 3·13) | 0·557 |
|  | Black | 1·99 (0·52 , 7·57) | 0·313 |
|  | Mixed | 0·20 (0·01 , 3·68) | 0·281 |
|  | Others | 2·05 (0·42 , 9·92) | 0·373 |
|  | Not stated | 1·62 (0·92 , 2·88) | 0·098 |
| Care home residence |  | 0·90 (0·55 , 1·47) | 0·666 |
| Mechanical ventilation |  | 1·55 (0·82 , 2·93) | 0·178 |
| ACEI or ARB use^ꙶ¥^ |  | 0·44 (0·25 , 0·76) | <0·001 |
| COVID-19 disease |  | 3·22 (2·24 , 4·62) | <0·001 |
| Charlson's comorbidity score | 0 | 1 (Ref) |  |
|  | 1 | 2.00 (1·12 , 3·56) | 0·018 |
|  | 2 | 2·19 (1·19 , 4·07) | 0·012 |
|  | 3 | 4·54 (2·54 , 8·12) | <0·001 |
|  | 4 | 4·36 (2·26 , 8·4) | <0·001 |
|  | 5+ | 6·72 (4·03 , 11·18) | <0·001 |
| Peak AKI | Stage 1 | 1 (Ref) |  |
|  | Stage 2 | 1·71 (1·03 , 2·81) | 0·037 |
|  | Stage 3 | 1·76 (1 , 3·08) | 0·049 |
| Hospital AKI |  | 1·18 (0·81 , 1·72) | 0·389 |
| AKI stage progression |  | 2·02 (1·14 , 3·58) | 0·016 |
| Renal replacement therapy |  | 1·87 (0·72 , 4·88) | 0·201 |

^¥^ Angiotensin converting enzyme or angiotensin receptor blocker
