# Supplementary material for: Acute kidney injury associated with COVID-19: A retrospective cohort study
Source: PLoS Med. 2020 Oct 30;17(10):e1003406. doi: 10.1371/journal.pmed.1003406 (PMC7598516; doi:10.1371/journal.pmed.1003406)
Supplement: Attachment — Submitted filename: Editors comments.docx [file pmed.1003406.s007.docx]

**EDITOR’S COMMENTS**

**ABSTRACT**

**Background- please revise “acute hospitals”**

*This has been rectified*

**Methods and findings- please provide participant demographics.**

*This has been added to the abstract*

**The last sentence of the methods and findings section must state 2-3 limitations of your work**

*This has been added to the abstract*

**AUTHOR SUMMARY**

*This has been added as requested*

**BIBLIOGRAPHY**

*Bibliography has been formatted as per Vancouver style*

**METHODS**

**KDIGO should be introduced on first view on page 5**

*KDIGO has been introduced on page 7, line 154*

**Please mention details of ethics approval and consent earlier in the methods section**

*Details of ethical permission has been introduced at the start of Method sectionon page 5-6, line 120-126*

**Did your study have a prospective protocol or analysis plan? Please state this (either way) early in the Methods section.**

*The study has a protocol with analysis plan and has been enclosed as S1 Text and is available as dx.doi.org/10.17504/protocols.io.bimskc6e*

**RESULTS**

**In the section “Factors associated with mortality in COVID-19” there seems to be an = sign missing in the third sentence and a p missing after “care home residents”. There are similar instances on Page 12,13
Please consistently provide p values of up to 3 decimal places, ensuring exact p values are provided unless p<0.001**

*The p values have been corrected to 3 decimal places and appropriate symbols have been inserted*

**DISCUSSION**

**Could you revise “Asians” to “of Asian ethnicity” ?**

*This has been corrected and line 392on page 17 reads as “Though a higher proportion of men and Asian ethnicity with COVID-19 had AKI,…”*

**Thank you for providing the STROBE checklist. Could I ask you to please provide this as a separate supplementary file and name it S1 Checklist. We also ask that the supplementary information is provided in individual files and referenced in text.**

*STROBE Checklist is enclosed separately as S2 Checklist and referenced on line 129 on page 6.*
